# Toxoplasmosis in an Immunocompetent Patient

**DOI:** 10.12669/pjms.346.15016

**Published:** 2018

**Authors:** Khalid Abdul Basit, Sadaf Nasir, Ejaz Vohra, Muhammad Kashif Shazlee

**Affiliations:** 1 *Khalid Abdul Basit, MBBS. Resident Medical Officer, Dr. Ziauddin Hospital, Karachi, Pakistan*; 2 *Sadaf Nasir MCPS (Internal Medicine). Registrar, Medicine Department, Dr. Ziauddin Hospital, Karachi, Pakistan*; 3 *Ejaz Vohra, FRCP (Edin). Chairman Medicine Department, Director of Postgraduate Training Program (Clinical), Former Principal Karachi Medical and Dental College, Dr. Ziauddin Hospital, Karachi, Pakistan*; 4 *Muhammad Kashif Shazlee, FCPS, EDiR(Paris), FRCR (London). Consultant Radiologist, Head of Radiology Department, Dr. Ziauddin Hospital, Karachi, Pakistan*

**Keywords:** Infection, Neurology, Toxoplasmosis

## Abstract

Toxoplasmosis is an obligate intracellular, food borne parasite disease with variable clinical presentation. Although the neurological presentation of toxoplasmosis in immunocompetent patients is uncommon, broad differential diagnosis should be kept in consideration when attending to similar patients. Twenty years old man with no known co-morbid conditions presented with fever and unilateral limb weakness for three weeks. It increased gradually, associated with altered level of consciousness for the last five days, diagnosed as acute toxoplasmosis. MRI Brain showed multiple ring enhancing lesions in frontal, parietal and temporal lobes. Serology for toxoplasmosis denoted raised IgM levels 36IU/mL (cut off value > 18IU/mL). This case report describes the clinical presentation and management of neurological toxoplasmosis in immunocompetent patient. Early diagnosis and prompt management can resolve the symptoms at an earlier stage.

## INTRODUCTION

Toxoplasmosis is an obligate intracellular, food borne parasite disease. It is transmitted through consumption of raw or uncooked meat contaminated by cat faeces, the definitive host of oocytes.^1^ Clinical presentation varies from asymptomatic or subclinical in healthy individual to neurological symptoms, ocular disease or superimposed opportunistic infections in immunocompromised.^2^,^3^ This paper presents a case of neurological toxoplasmosis in immunocompetent individual.

## CASE PRESENTATION

A 20 years old man with no known co-morbid conditions presented with low grade fever and unilateral limb weakness for three weeks which increased gradually, associated with altered level of consciousness for the last five days. Rest of the history was unremarkable, except that he had positive contact history of pets.

### Examination

On inspection ill looking young thin lean man was irritable and confused on verbal response. General physical examination revealed blood pressure of 125/80, pulse was 95 per minutes, respiratory rate was 22 breaths per minute and temperature was 39°C. Neurological exam showed Glasgow coma scale of 13/15 (E4, M5, V4). Neck stiffness was positive. Increased tone was noted in right lower limb, while bulk was normal and equal bilaterally. Power was decreased in right upper and lower limbs, measuring 1/5, while it was 5/5 in left upper and lower limbs. Planters were up going bilaterally and pupils were reactive to light in either eye.

### Hospital course

Initial laboratory investigations included complete blood count, urea, creatinine and electrolytes, liver function tests, calcium, magnesium and albumin, all were within normal limits. Lumber puncture showed protein of 46mg/dl (20-40mg/dl), glucose of 72mg/dl (60% of plasma glucose), 6 RBCs (0-4/cumm) and 5 white blood cells (Nil). Blood culture, CSF culture and PCR were negative. MRI brain showed multiple ring enhancing lesions in white and grey matter involving corpus callosum, subcortical areas and periventricular region in frontal, parietal and temporal lobes. The lesions were surrounded by vasogenic edema appreciated on coronal FLAIR image. AFB smear and MTB DNA were negative. C3 (146.5) (normal range 90-180) and C4 (26.1) (normal range10-40) levels, done to rule out hypocomplementemia and were within normal limits. The ratio of CD4:CD8 was within normal range (0.98) (reference value 0.68-2.73). Toxoplasmosis IgM levels 36IU/mL (normal value<18 IU/mL) and IgG levels were raised 57.7IU/mL (normal range ≤ 8 IU/mL). Anti-HIV antibody test by CMIA method and HIV core protein p24 were negative. (Reference ranges>1.0).

**Fig.1:**
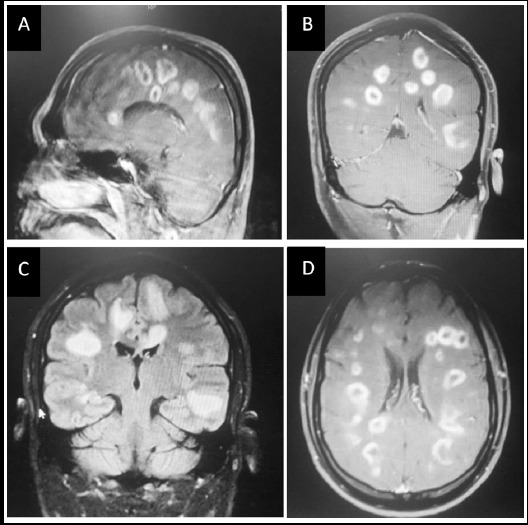
Contrast enhanced MRI T1 weighted images showing multiple ring enhancing lesions in white and grey matter of supratentorial region bilaterally.

He was treated with combination therapy of trimethoprim / Sulfamethoxazole and folic acid. Due to unavailability of 1^st^ line treatment i.e. pyrimethamine and sulphadiazine in the country above mentioned drugs were used. IV steroid were used to subside the inflammatory reaction. Subsequent MRI after two weeks showed reduction with size of the lesions with decrease in surrounding edema.

## DISCUSSION

Infection of humans with Toxoplasmosis Gondii are becoming prevalent worldwide, depending on the environment, eating habits and age.^4^ Toxoplasmosis Gondii is an obligate intracellular protozoal parasite and it is of three types:

1. The tachyzoite,
2. Bradyzoite,
3. Sporozoite,^5^

In one of the previous study conducted in U.S in 2009, Jones revealed that high risk of infection caused by Toxoplasmosis Gondii was due to the following risk factors: eating raw ground beef, eating locally produced curd, dried, or smoked meat, eating rare lamb, working with meat, drinking unpasteurized goat milk, eating raw oysters, clams, or mussels.^6^ Previous case reports also mentioned that the prevalence among men is more than women (79% versus 63.4%).^4^

**Fig.2:**
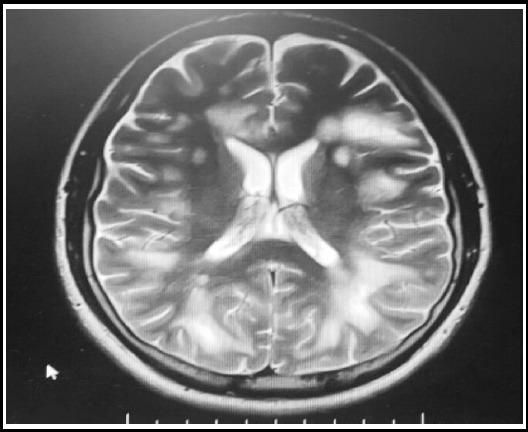
T2 weighted axial image showing decrease in size of multifocal white matter lesions with perilesional edema after treatment initiation.

Clinical manifestation of the disease caused by Toxoplasmosis Gondii relies upon the age and immune status of the patient.^7^ Immunocompetent individuals are usually asymptomatic in the acute phase of infection. However, a latent phase occurs and is associated with persistence of the organisms primarily in the heart, skeletal muscles, and brain. The most common presenting symptom in patients is headache and is usually accompanied by fever and altered mental status. Patients may also present with seizures, cranial nerve abnormalities, visual field defects, and sensory disturbances. Common focal neurological signs include motor weakness and speech disturbances.^8^ The most often damaged areas in central nervous system (CNS) include the basal ganglia, corticomedullary junction, white matter, and periventricular regions.^5^

Several methods are used for diagnosis of Toxoplasmosis Gondii infection but gold standard tests are enzyme-linked immunosorbent assay (ELISA) and indirect immunofluorescence assay (IFA) for detection of Toxoplasma-specific antibodies (IgG or IgM).^9^ MRI is the best initial screening radiological procedure for CNS toxoplasmosis. Typical radiological findings are bilateral, multiple, ring-enhancing lesions over basal ganglia and corticomedullary junctions of the cerebral hemisphere. However approximately 14% of cases, the lesions are solitary. Hemorrhage may be seen occasionally, which is used to differentiate toxoplasmosis from lymphoma.^7^

Treatment with pyrimethamine, sulfadiazine and folic acid is used for patients who are immunocompromised and those immunocompetent patients with severe or persistent symptoms.^4^ Short course of corticosteroids can be used in toxoplasmosis encephalitis patients with significant cerebral edema and elevated intracranial pressure. Toxoplasmosis will reoccur if therapy is discontinued.^10^ It is essential to rule out differentials like primary CNS lymphoma, tuberculosis, pyogenic, and fungal infections.^11^

## CONCLUSION

Infections caused by Toxoplasmosis Gondii are more frequently seen in immunocompromised patients such as individuals suffering from HIVAIDS but it can occur even in immunocompetent individuals, as this case revealed. A broad differential diagnosis should be kept in consideration, early detection and appropriate intervention can cure the disease. The risk factors of disease should be discussed in detail to reduce future acquisition of infection.

### Author’s Contribution

**KAB:** Literature search, wrote and approved the final submitted version.

**SN:** Concept, design, Clinical management and diagnosis, edited and approved the final submitted version.

**EV:** Concept, design, Clinical management and diagnosis, edited and approved the final submitted version.

**MKS:** interpretation of Radiological data, edited and approved the final submitted version.
